# Three-Phase Energy Model for Surface Fracture Mechanisms in Cutting Soft Tissues with Snare-Type Tools

**DOI:** 10.1007/s10439-026-04029-5

**Published:** 2026-02-16

**Authors:** Jinghang Wang, Urara Satake, Toshiyuki Enomoto

**Affiliations:** Division of Mechanical Engineering, Graduate School of Engineering, The University of Osaka, 2-1, Yamada-oka, Suita, Osaka 565-0871 Japan

**Keywords:** Snare-type tools, Soft tissues, Surface fracture, Energy model, Friction dissipation

## Abstract

**Purpose:**

Snare-type tools are widely used for polyp tissue resection in cold snare polypectomy. Due to the characteristics of snare-type tools, which fracture the inner tissue first and the surface later, high friction in the contact area may hinder the extension of cracks to the surface, resulting in a lower-quality cut and an increased risk of bleeding. This study is to investigate the mechanism of tissue surface fracture and give guidance on the optimization of tools and techniques.

**Methods:**

This paper proposes a three-phase energy model to explain the mechanism of soft tissue surface fracture. The energy changes in these three phases are characterized by experiments and finite element simulations. The factors affecting the cutting ability of the tool and the surface fracture state are further explored through single-factor experiments.

**Results:**

The results show the effectiveness and consistency of cutting fracture toughness and section root-mean-square (RMS) height as indexes for evaluating the cutting ability and surface fracture state of snare-type tools. High cutting speeds can increase cutting fracture toughness and reduce the RMS height, and surface lubrication reduces the RMS height, but wire diameter has no significant influence on them.

**Conclusion:**

This study introduces a novel energy-based model for clearing the understanding of surface fracture mechanisms for the field of soft tissue cutting and provides guides for the optimization of snare-type tools and surgery technologies.

## Introduction

Soft tissue cutting is widely used in the medical and biological fields, and cutting efficiency and safety are the research focus [1, 2]. Snare-type tools are used to accomplish polyp tissue cutting in cold snare polypectomy (CSP). Unlike conventional surgical tools, it captures and cuts soft tissue through a wire rope [3]. A recent study has identified the cutting mechanism by which tissue starts to fracture from the inside when cut with snare-type tools, and the factors affecting the maximum cutting force have been investigated based on cutting safety [4]. It was also found that high friction in the contact area may hinder the extension of cracks to the surface, resulting in a lower-quality cut and an increased risk of bleeding [5]. The cutting process is usually divided into three phases: deformation, fracture, and cutting in soft tissue cutting research [6]. Most studies focus on the stability of the cutting phase or view the three phases as an overall puncture problem [7]. However, during the cutting process with snare-type tools, the large contact area between the snare-type tool and the tissue increases the energy required for fracture and slows down the fracture process, resulting in the peaks and variations of the cutting force concentrated between the deformation and the fracture phases, which is difficult to observe with conventional tools and result in lacking the study of this process. Therefore, understanding the mechanism of the surface fracture process is critical for solving the surface fracture problem and optimizing snare-type tools and technologies.

Some studies have shown that friction may play a significant role in the surface fracture process. Liu et al. pointed out through finite element simulation that the friction between the blade and the tissue results in the inability to produce large deformation of the tissue directly below the contact surface, and the place of the maximum strain where cracks may be produced is located at a certain distance below the tissue, which is an essential factor in preventing the tissue from fracturing [8]. Satake et al. further analyzed the relationship between friction and the maximum stress state at the beginning of cutting through simulation and determined a strong correlation [9, 10]. However, measuring surface friction during cutting is difficult, so most studies can only be performed based on simulation software. Compared with the direct analysis of the cutting force, the energy point of view can be integrated to explore the fracture, strain, friction, and other influencing factors[11], suitable for applications in the occasion of snare-type tool cutting, such as large deformation and friction force are difficult to measure.

Traditional fracture energy models are primarily used to study the efficiency and stability of the cutting or the puncture resistance of materials [12, 13]. Meanwhile, friction dissipation during cutting is usually ignored or separated by special methods [14]. Hu et al. used a quasi-static fracture model to investigate the cutting process, which did not consider the friction [11]. Atkin et al. analyzed the effect of the slice/push ratio using an energy relationship that takes friction into account, but only for the stable cutting process [15]. Triki et al. studied the fracture energy of cutting glove membranes based on the theory of Rivlin and Thomas [16]. For snare-type tools, not only the energy changes of the surface fracture process need to be specifically analyzed, but also the effect of friction dissipation cannot be ignored. Therefore, the traditional energy equation is unable to explain the energy change.

To address these challenges, a new fracture energy model is urgently needed to describe the surface fracture process of tissue. Meanwhile, appropriate indexes are needed as a basis for improving surface fracture performance and snare-type tools optimization.

This paper proposes a three-phase energy model to explain the surface fracture mechanism in cutting soft tissues with snare-type tools. Furthermore, cutting fracture toughness and section RMS height were proposed for evaluating the cutting ability of the tool and surface fracture state of the tissue, and their validity and influencing factors were investigated through single-factor experiments. This study clears the understanding of surface fracture mechanisms for the field of soft tissue cutting and provides guides for the optimization of snare-type tools and surgery technologies.

This study is an extension of our previous study, so part of the experimental setup and material selection are the same to ensure the continuity and comparability of the experimental results. While our previous study established the fundamental fracture mechanism of snare-type tools [4], this study extends the investigation to the critical surface fracture phenomenon that directly influences the surgical cutting performance, and proposes evaluation indexes and optimization directions for the cutting ability of snare-type tools.

## Materials and Methods

### Surface Fracture Process and the Energy Model

Based on the traditional quasi-static fracture energy model and the difference in energy conversion [17], the surface fracture process is divided into three phases:

- Phase 1: Elastic deformation:1
- Phase 2: Crack generation and propagation:2
- Phase 3: Overcoming friction:3where ${W}_{c}$ is all the work done by the external force. ${W}_{s}$ is the strain energy stored in the material. $J$ is the cutting fracture toughness. $dA$ is the fracture area. $JdA$ is the fracture energy consumed when the crack generates and expands. ${W}_{f}$ is the energy required to overcome friction or frictional dissipation, and Ґ represents the inelastic strain energy dissipation from stress relaxation and viscoelastic deformation.

**Fig. 1:**
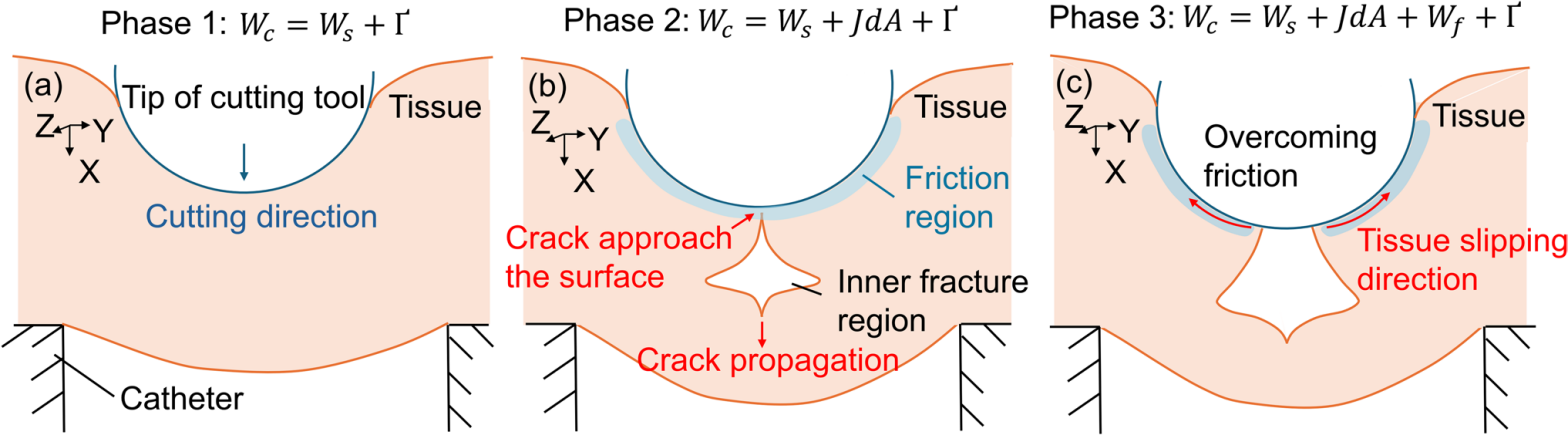
An ideal surface fracture process of cutting with snare-type tools

Figure 1 demonstrates an ideal surface fracture process. During Phase 1, the work done by the tool ${W}_{c}$ is completely converted into strain energy ${W}_{s}$ of the tissue and stored (Fig. 1a). With the internal tissue approaching the strength limit, the strain energy stored in the tissue creates conditions for crack initiation which is partially converted into the fracture energy $JdA$ (Fig. 1b). The crack is compressed in the X-direction and stretched in the Y-direction, extending along the X-direction to the tissue surface. When the crack closes to the surface, it is necessary to overcome the friction between the tissue and the tool surface to make them produce relative slip (Fig. 1b, c), which is supported by the stored strain energy. If the fracture is realized successfully, the strain energy will be converted to frictional dissipation ${W}_{f}$ (Fig. 1c), resulting in an instantaneous reduction in the cutting force.

${W}_{c}$ can be obtained from the integral of the cutting force ${F}_{c}$ in the direction of displacement:

4 $${W}_{c}={\int }_{0}^{x}{F}_{c}(x)dx$$

The strain energy ${W}_{s}$ is difficult to calculate directly in the case of large deformations but can be separated by multiple loading experiments.

The energy ${W}_{f}$ required to overcome friction can be calculated by:

5 $${W}_{f}=2\times {\int }_{0}^{l}{F}_{f}(l)dl=2\times {\int }_{0}^{l}\mu {F}_{N}(l)dl$$

${F}_{f}(l)$ is the distribution of friction on the contact surface, $\mu$ is the friction coefficient, and ${F}_{N}(l)$ is the distribution of normal force on the contact surface, $l$ is the contact length.

The cutting fracture toughness $J$ of the material can be given by Eqs. (2, 3):67

Previous studies calculating cutting fracture toughness have generally used Eq. 7 because they focused only on the beginning and end states of the cut. However, due to the difficulty of calculating frictional dissipation, it is generally solved using special Y-cutting methods or lubrication, which are cumbersome to practice and introduce additional errors [14]. In this study, the cutting fracture toughness can be calculated by the area of the inner fracture region using Eq. 6, which could avoid the effect of surface friction.

From Eqs. 2 and 3, the work done by the external force ${W}_{c}$ in Phase 2 is mainly related to the fracture energy $JdA$, which in Phase 3, is related to the fracture energy $JdA$ and the frictional dissipation ${W}_{f}$. The cutting fracture toughness $J$ can be expressed as the energy required to fracture a unit area of material, so the smaller $J$ is, the less energy is required, and the easier for the tool to cut the material. Therefore, $J$ can be regarded as a performance of the cutting ability of the tool, determining the consistency and influence factors of $J$ is critical. For Phase 3, there are two situations. The first one is that the tissue in the contact area overcomes friction and fractures smoothly, as shown in Fig. 1. In this case, the required fracture energy is small. However, in some cases of large deformation, the friction on the contact surfaces is high, resulting in an inability to overcome the surface friction to fracture the surface of the contact area, which transfers the fracture to the non-contact area [4]. In this case, the fracture energy will be much larger. Therefore, the work done by the external force in Phase 3 mainly depends on the surface fracture state. The evaluation index of the surface fracture state and the influencing factors are especially critical.

The energies in the model and their variations will be characterized by experiment and simulation. The cutting fracture toughness $J$ and surface fracture state, as the critical factors affecting energy, will be investigated by single-factor experiments.

### Finite Element Simulation

Since friction is difficult to measure directly, the distributions of friction and influencing factors were investigated using finite element simulation.

**Fig. 2:**
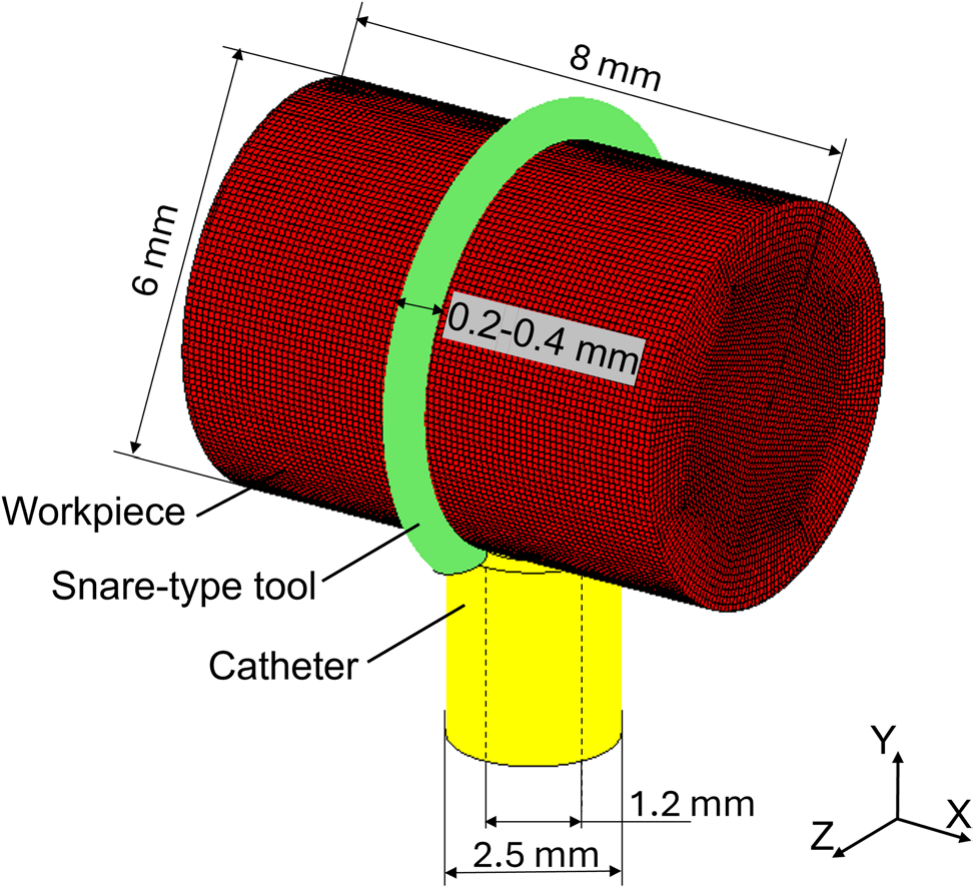
Schematic diagram of finite element simulation model

The commercial finite element software package MSC Marc/Mentat (MSC Software, Version 2024.2) was used in this study. The 3D finite element model consists of a snare-type tool, a workpiece and a catheter, which is shown in Fig. 2. The workpiece is a cylinder with a length of 8 mm and a diameter of 6 mm, and the catheter has an outer diameter of 2.5 mm and a inner diameter of 1.2 mm. To ensure the accuracy of the numerical calculations and considering that too fine a mesh would increase the calculation cost, the mesh on the surface was refined to 10 μm. The material used was the Mooney-Rivlin model, which is commonly used in the simulation of bio-soft tissue materials, and the parameters used in the model come from the properties of the arterial wall, C1 = 0.174 MPa and C2 = 0.145 MPa [18].

Different radii of wires and friction coefficients were set to collect the distribution of friction force and the total energy required to overcome the friction force under different indentation depths, respectively. The practical use of the commercial snare usually has diameters of 0.2–0.4 mm, so the wire diameters are set to be 0.2, 0.3, and 0.4 mm, which are just in contact with the workpiece in the initial state, and no interaction force is generated. The friction coefficient between the workpiece and the wire is set to be 0.1–0.5. Since too large a deformation of an element can make the calculation difficult to converge, setting the maximum indentation depth to 2–3 times the diameter of the tool allows the trend to be observed without exceeding the limits of the arithmetic ability. The tool moves along the Y-axis with a total distance of 1 mm under constant velocity.

### Fracture Energy Characterization Experiment

Since fracture, strain and friction energies are coupled with each other during the cutting process, it is necessary to differentiate and characterize the energies of each phase through cutting force curves.

The material of the tissue sample and the mechanical validation followed the method in our previous study [4], where silicone rubber (Ecoflex 00-10) was used and calibrated to match human colonic tissue properties by incorporating silicone oil (090-50CS, AZ) [19].

**Fig. 3:**
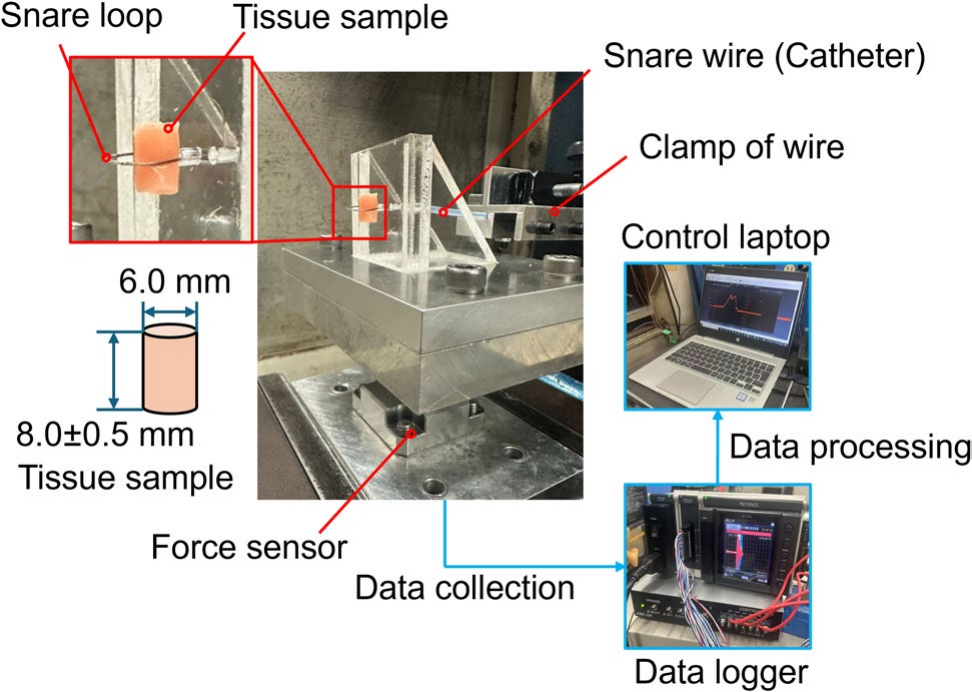
Experimental setup of the fracture energy characterization experiment

The experimental platform followed our previous setup (Fig. 3), comprising a three-axis machining center (AJV-18, Yamazaki Mazak), a force sensor (9327C, Kistler), and a data logger (NR-600, Keyence) [4]. The cutting of the snare device is imitated by controlling the X-axis movement of the machining center. The force sensor monitors the change of cutting force during the cutting process in real time and collects and transmits it to the control laptop through the data logger. Tissue samples were made into cylindrical shapes with a diameter of 6.0 mm and a length of 8.0 ± 0.5 mm to reduce the effect of stress concentration.

The first experiment was used to differentiate the energy model by the whole-cycle cutting force curve, tissue samples were completely cut using a snare-type tool, and data of the whole cutting process were recorded. The second experiment was conducted by loading-unloading-loading. Pause the tool when the cutting force reaches the first peak (inner fracture) and wait for the cutting force to decrease until it stabilizes, making sure that the strain energy is fully converted into fracture energy. Then, record the stabilized cutting force and unload the tool. The second time, the cutting force is loaded to the recorded stabilized state, and the cutting force curves for both loadings are recorded. After cutting, the $A$  region 1 of the central fracture region on the tissue cross-section is measured using a laser microscope (EVK-X200, KEYENCE) and data analysis software (VK-H1XMC, KEYENCE), and the cutting fracture toughness is calculated by Eq. 6.

### Surface Fracture State Influence Factor Experiment

The snare-type tool generates a large strain when cutting soft tissues, which makes it difficult for the finite element simulation calculations to converge. Thus, it is impossible to determine whether the tissue surface is smoothly fractured. As seen from Fig. 1, when the surface is smoothly fractured, the cracks are generated from the inner part of the tissue and expand to the surface along the X-direction with the stretching in the Y-direction, so the cross-section should be flat and integrated. However, when the surface cannot fracture smoothly, resulting in the fracture area being outside the contact area, the cross-section will be uneven. Therefore, the surface flatness will be different due to the effect of the surface fracture state. Single-factor experiments were conducted to verify the difference in the surface flatness and to investigate the factors affecting the surface fracture state.

The variables studied in the experiments were the cutting speed and lubrication conditions between the snare and the tissue. In the cutting speed experiments, cutting speeds were set to be 50, 100, 200, 300, and 400 mm/min. In the lubrication condition experiments, three conditions were performed: no lubrication, water lubrication, and oil lubrication. In the lubricated condition, the tissue samples and the tip of the snare were immersed in pure water and silicone oil for 1 min in advance to change the coefficient of friction of the contact surfaces. To ensure the generalizability of the experimental results, 5 typical commercial snares with different morphological characteristics were selected (Table 1). All the above experiments were carried out at room temperature, with each set of experiments repeated three times to estimate the repeatability results were averaged for subsequent analysis.

**Table 1:** Commercial snare devices used in the cutting experiments

No.	Manufacturer	Type specification
Snare1	PENTAX	VDK-SD-23-230-20-A1P
Snare2	PENTAX	VDK-CS-23-230-15-C3P
Snare3	PENTAX	VDK-SD-23-230-15-A1P
Snare4	OLIMPUS	SD-230U-20
Snare5	OLIMPUS	SD-210L-15

## Result

### Distribution of Interfacial Friction

**Fig. 4:**
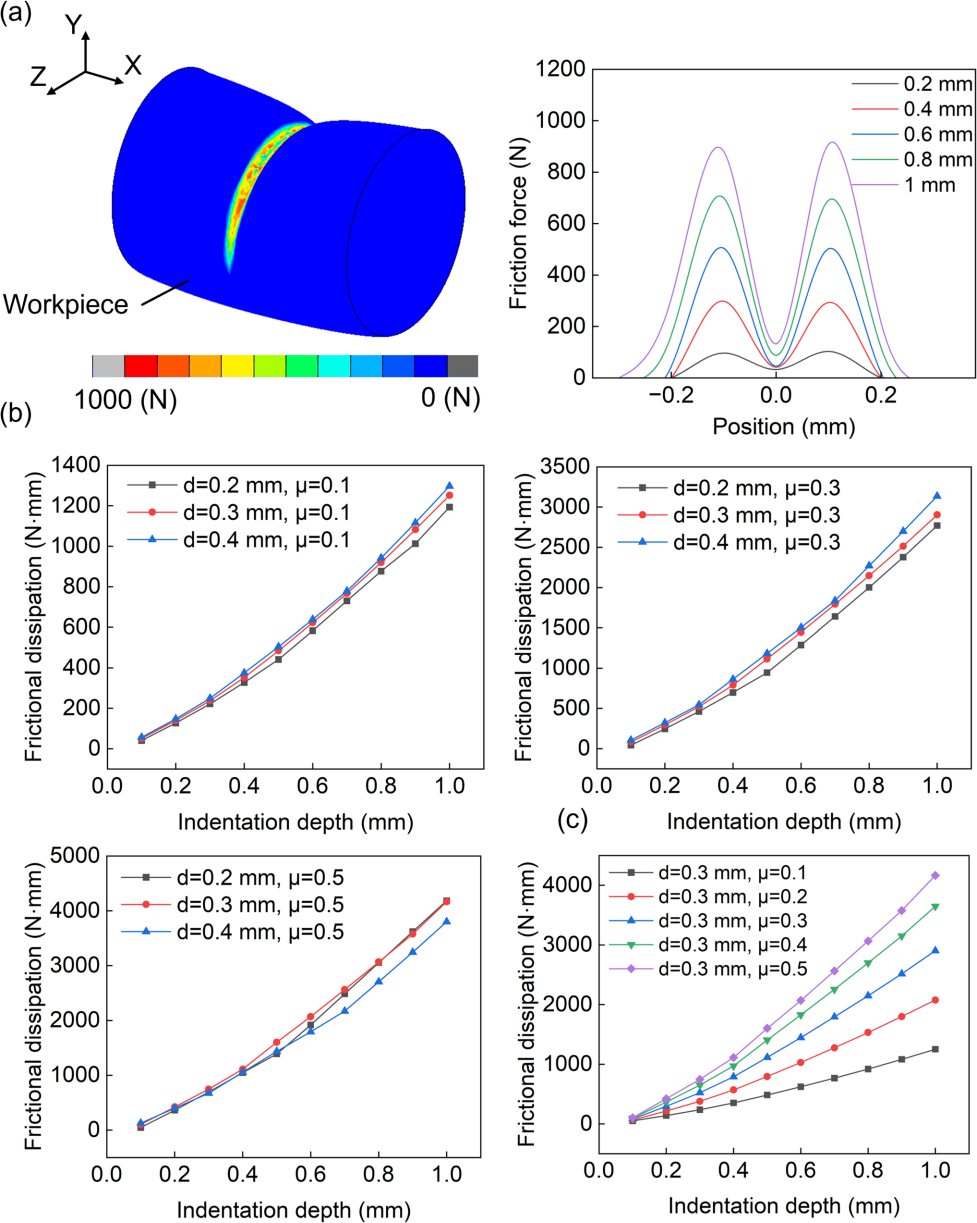
Finite element simulation results. **a** Schematic of the simulation results and the friction distribution on the contact surface; **b** Friction dissipation variation with tool diameters; **c** Friction dissipation variation with friction coefficients

Collecting the finite element simulation data in a rectangular region with a length of 1.1 mm and a width of 0.7 mm at the center of the contact surface between the wire and the tissue. The friction distribution change of the workpiece center point by X-axis, with indentation depth of 0.1–1 mm, a wire diameter of 0.3 mm, and a friction coefficient of 0.3, is shown in Fig. 4a. The friction force is small at the center point and increases first, then decreases with deviation from the center position. The maximum friction force and the distribution range increase with indentation depth. The surface frictional dissipation is calculated by Eq. 5, and the variations of frictional dissipation with  indentation depth for different tool diameters and friction coefficients are shown in Fig. 4b, c. Within 1 mm indentation depth, the friction dissipation increases with increasing indentation depth. At the same friction coefficient, the wire diameter has a small effect on the friction dissipation (Fig. 4b). For the same wire diameter, the friction dissipation increases with the increase of the friction coefficient, but the extent of increase decreases with the increase of the friction coefficient (Fig. 4c).

### Cutting Fracture Toughness

**Fig. 5:**
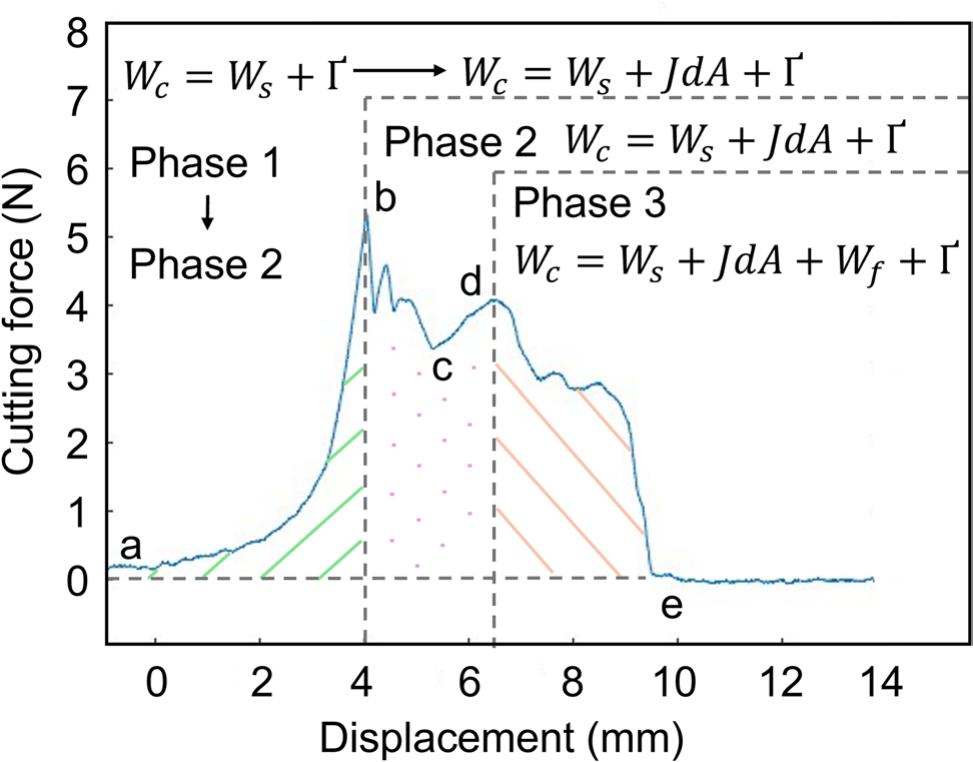
Change of cutting force with tool displacement

The cutting force curve for a whole-cycle cutting with Snare1 under 100 mm/min cutting speed is shown in Fig. 5, where the tool starts contacting the tissue surface at point a, and continues until the tissue is completely fractured and separated from the tool at point e. During the process of a–b, the surface fracture is transformed from Phase 1 to Phase 2. According to a previous study, roughly from cutting force is about 3 N (Cutting speed 100 mm/min), cracks will start to appear inside the tissue, and with the increase of cutting force, the internal cracks gradually expand [4]. After reaching point b, the cracks within the tissue connect to form a fracture, and most of the strain energy is released. With the tool continuing to move, the remaining strain energy within the outer tissue accumulates again. When the cut reaches point d into Phase 3, the residual tissue fractures, strain energy is converted into fracture energy and friction dissipation, and the crack gradually expands until the tissue is completely cut off.

**Fig. 6:**
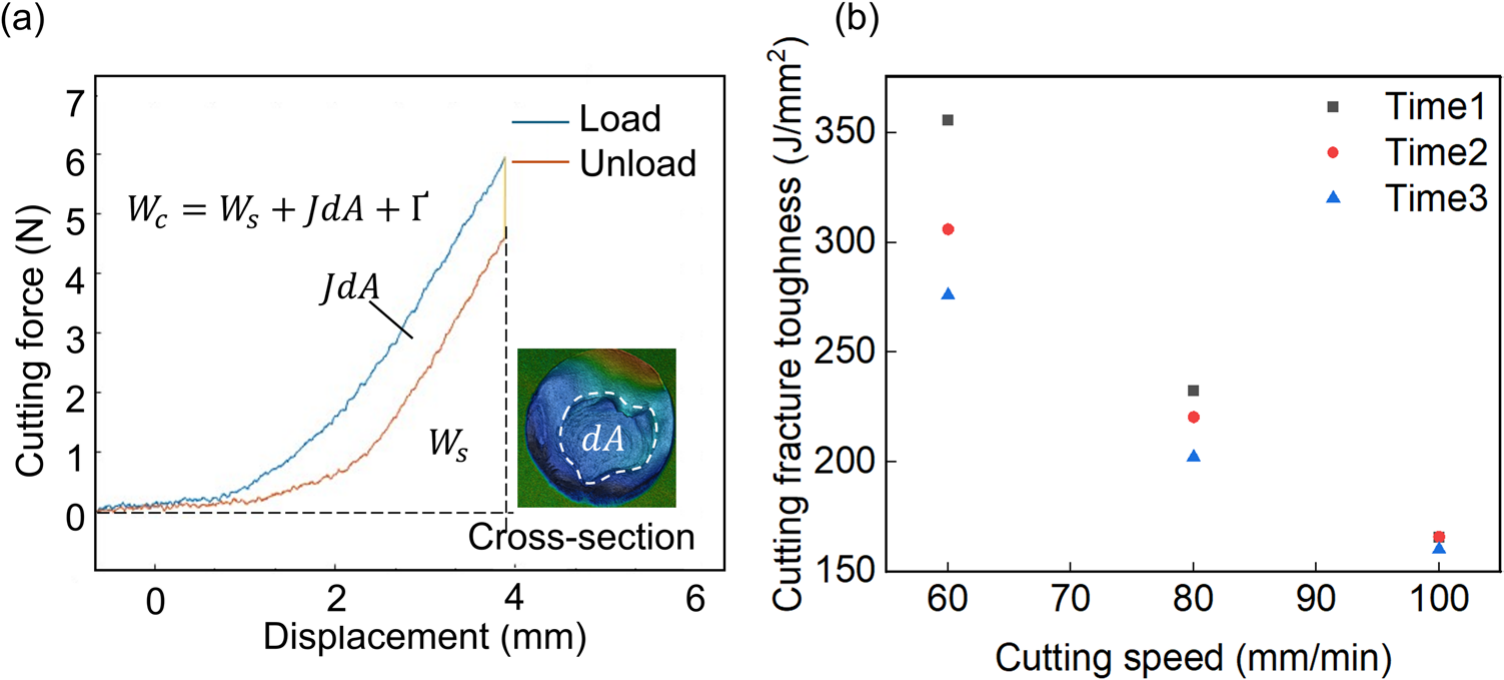
Cutting fracture toughness calculation: **a** loading-unloading-loading curve and fracture area calculation; **b** cutting fracture toughness distribution with cutting speed

The fracture energy in Phase 2, separated by secondary loading, is shown in Fig. 6a. The upper region area is the consumed fracture energy $JdA$, and the lower region area is the residual strain energy ${W}_{s}$. The area $A$ of the central fracture region on the tissue cross-section is measured, and the cutting fracture toughness can be calculated by Eq. 7.

To verify the consistency of the cutting fracture toughness, experiments were conducted under different cutting speeds, and the results are shown in Fig. 6b. The differences in cutting fracture toughness between different cutting speeds were significant and have a high reproducibility, proving that the cutting fracture toughness is effective. Cutting speed and cutting fracture toughness have a strong correlation, the higher the cutting speed, the lower the cutting fracture toughness. Meanwhile, in the case of low cutting speed (60 mm/min), the consistency of the cut fracture toughness obtained from the experiments is low, while the consistency gradually becomes higher with the increase of cutting speed, and the cutting fracture toughness obtained from the three times are almost the same at 100 mm/min.

### Characterization of Surface Fracture State

**Fig. 7:**
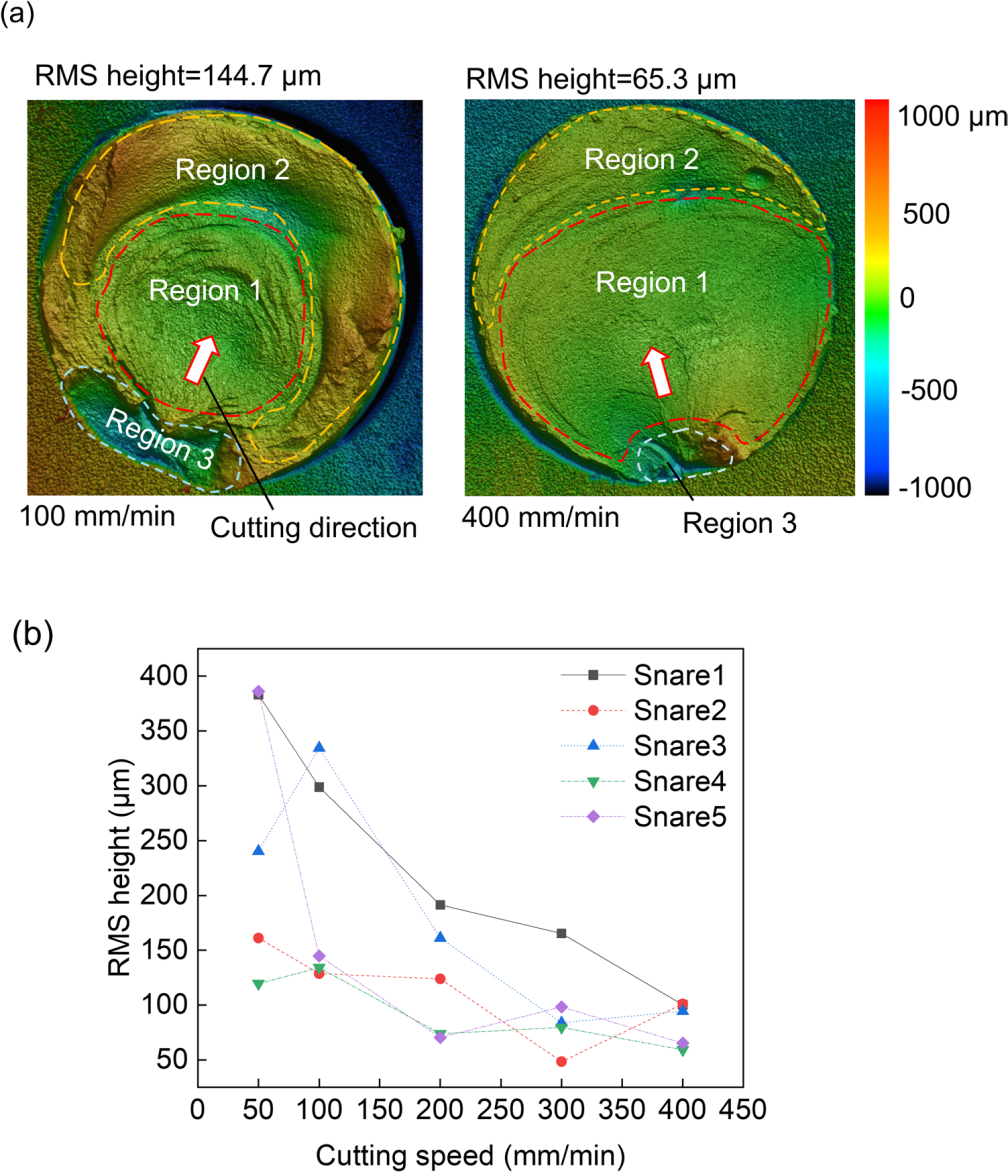
Relationship between RMS height of cut section and cutting speed: **a** Cut section morphology; **b** RMS height variation with cutting speed

Fig. 7a shows the cross-sectional morphology of the tissue samples after cutting at 100 and 400 mm/min speeds, respectively, using Snare5. It can be found that the center region 1 is flat when the cutting speed is 100 mm/min, while region 2 is higher than region 1, and region 3 is lower than region 1, indicating that the fracture of these two regions is not continuous and smooth. At 400 mm/min, the center region 1 and the outer region 2 are almost in the same plane, and only region 3 has obvious unevenness in a small region. Taking the plane of the center flat region as an index, which is represented by the root-mean-square (RMS) height, the RMS height is 144.7 μm at 100 mm/min speed and 65.3 μm at 400 mm/min speed, which is a significant difference.

Fig. 7b shows the RMS height at different cutting speeds using five different commercial snares. The RMS height of all snares decreases gradually as the cutting speed increases. However, the cutting results of different snares varied greatly at low speeds. At a speed of 50 mm/min, the RMS heights of Sanre1 and Sanre5 were large, reaching about 400 μm, while Sanre4 and Sanre5 were smaller, at about 150 μm. At speeds of up to 400 min/min, the RMS height of all snares had RMS heights that decreased under 100 ± 25 μm. Based on the decreasing trend of RMS height, it can be predicted that as the cutting speed continues to increase, the RMS height will stabilize in the range of 50–100 μm, which can be regarded as the limiting threshold for the surface flatness of soft tissue cut by snare-type tools.

**Fig. 8:**
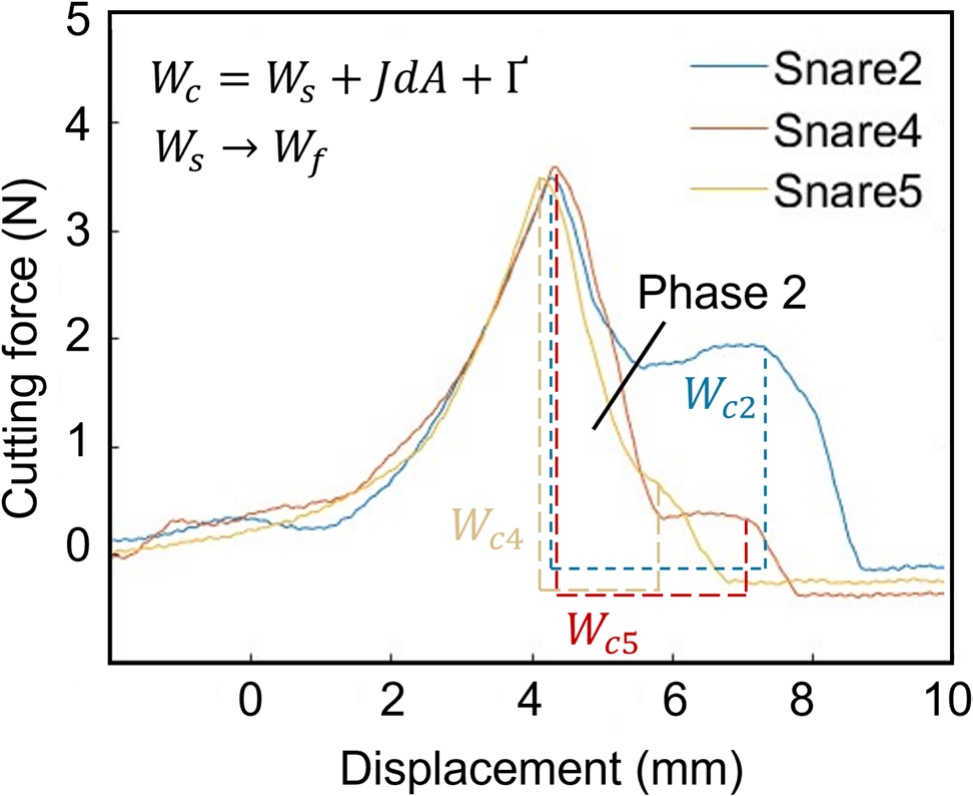
Friction energy comparison of Snare2, Snare4, and Snare5

To verify the validity of the RMS height, Snare4 and Snare5, at a speed of 400 mm/min, which have a small RMS height, and Snare2 (< 100 μm), which has a larger RMS height (> 100 μm), are taken for energy characterization experiments. The variation of cutting force with time is shown in Fig. 8. It can be found that in the case of small RMS height, after the force peak (inner fracture), the cutting force does not rise much as the cutting proceeds, less strain energy is stored in the tissue, and the surface fracture can be achieved quickly. When the RMS height is larger, more strain energy is stored within the tissue, and the cutting force rises for a period before fracture occurs. The displacement in Phase 2 is integrated with the cutting force to obtain the work done by the external force for three snares: ${W}_{c2}=0.1269 N\cdot m$, ${W}_{c4}=0.0716 N\cdot m$, and ${W}_{c5}=0.0657 N\cdot m$. The energy required for surface fracture in the case of small RMS height is much smaller than that in the case of large RMS height, which can be matched with the model that the frictional dissipation from overcoming friction when the surface can be fractured smoothly is smaller than the fracture energy required for fracture in non-contact regions. It can demonstrate the effectiveness of the RMS height of the cross-section as an index of surface fracture state, and the RMS height being less than 100 μm can be assumed as an index of a smooth surface fracture.

The cutting speed is closely related to the surface fracture state, and the higher the cutting speed, the easier it is to achieve a smooth surface fracture. Different types of commercial snare have different abilities in achieving smooth surface fracture. Snare5 has poor performance at low speeds, but the effect improves significantly as the speed increases and ultimately achieves smooth surface fracture. Snare2 and Snare4 have better fracture performances at low speeds but are unstable. With Snare1 and Snare3, although the fracture performance is improved with increased cutting speed, it still cannot realize the surface smooth fracture at 400mm/min.

Table 2 shows the RMS height of the section after cutting with Snare5 under different lubrication conditions at a cutting speed of 400 mm/min. The mean value of RMS height is 82.140 μm for no lubrication, 60.923 μm for water lubrication, and 67.115 μm for oil lubrication. Although the RMS height was overall lower in the case of lubrication than in the case of no lubrication, it can be seen from the results of the three experiments that the improving effect is not stable, and sometimes the RMS value of the cutting results in the no lubrication condition can reach the performance of the lubrication effect.

**Table 2:** Surface RMS height of different lubrication states

RMS height (μm)	No lubrication	Water lubrication	Oil lubrication
Time 1	93.248	58.010	80.043
Time 2	64.850	63.222	61.066
Time 3	88.323	61.538	60.236

## Discussion

### Cutting Fracture Toughness

Compared to our previous study that used the maximum cutting force as an evaluation index for the cutting ability of snare-type tools [4], which is also used in most of the related researches [20, 21], the new energy model not only describes the whole cutting process, but also quantifies the cutting ability of the snare-type tools in a more profound way through the cutting fracture toughness. Cutting fracture toughness is suitable for describing the difficulty of the cutting process or the cutting capacity of the tool. It is not a material constant but is affected by cutting parameters, tool structure, and material inhomogeneities and inconsistencies [22, 23]. For snare-type tools, a lower cutting fracture toughness represents a larger fracture area inside the tissue in Phase 2, under the same fracture energy and material properties. It will result in a lower fracture energy required for the fracture in Phase 3, reducing the difficulty of a smooth surface fracture. It can be observed from Fig. 6b that at low cutting speeds, the results of cutting fracture toughness exhibit poor consistency. It is caused by the higher cutting toughness, which results in higher and unstable inelastic strain energy dissipation [11]. Therefore, a reduction in the cutting fracture toughness implies not only an increase in the whole-cycle cutting capacity of the snare-type tool but also an increase in the stability of the cutting results.

### Surface Fracture State

A smooth surface fracture is not always achieved during the snare-type tools cutting soft tissue. In the case of smooth fracture, the energy required to overcome friction is low to begin with, fracture can be achieved without accumulating much strain energy (Fig. 8). On the contrary, when the friction is high to begin with and grows rapidly as the cut proceeds, the growth of the strain energy cannot satisfy the requirement of the growth of the energy for overcoming friction, resulting in the contact area never being able to fracture. When the increased strain energy is sufficient to provide the required fracture energy in the non-contact region, the non-contact region will fracture before the contact region, so the new fracture surface is not integrated with the fracture region inside the tissue, resulting in a high RMS height of the cross-section. After the tissue surface fracture, when the tool cuts the lower tissue, frictional dissipation is also generated by the contact of the tool with the inner surface of the tissue. Although the mechanism of tissue fracture is more complex in this phase, the effect of friction is the same, and the results can also be reflected in the RMS height, so the analysis will not be repeated. In a word, RMS height can be used as an index to evaluate the surface fracture state. In the optimization of the snare-type tool, efforts should be made toward reducing the RMS height of the cross-section.

### Influence Factors of Surface Fracture State

As an important parameter in the cutting process, cutting speed is independent of cutting fracture toughness in previous needle puncture studies [22], or has a non-significant effect on the cutting force [4]. In this study, cutting speed was found to be strongly correlated with the whole-cycle process of soft tissue cutting with snare-type tools. Firstly, an increase in cutting speed decreases the cutting fracture toughness of internal tissue. Due to the viscoelastic properties of soft tissue materials, in a short period, the local deformation concentrates near the contact area, while the overall deformation of the tissue is small, which can promote the fracture nucleation in the local contact area [24]. Secondly, as the fracture area within the tissue becomes larger and the area of residual tissue becomes smaller, the fracture energy decreases, and more energy is available to overcome surface friction. Therefore, the increase in cutting speed makes it easier to accomplish a smooth surface fracture.

Reducing the friction coefficient of the contact surfaces can reduce the energy required to overcome the surface friction, and thus decrease the difficulty of smooth fracture. However, the difference in cutting performance between the water and oil lubrication conditions in the experimental results is not obvious and unstable. This may be caused by the discontinuity of the water or oil film formed due to the pressure on the contact surfaces. At the same time, to ensure safety, the lubrication method does not apply to actual surgery. Therefore, in practical applications, friction should be reduced by other methods, such as changing the tool structure.

There is no significant relationship between the tool wire diameter and the frictional dissipation. Although smaller tool diameters can reduce the contact area between the tool and the tissue, it may result in greater strain and stress concentration, which increases the friction per unit area, making it unable to reduce the frictional dissipation on the contact surface. However, it is undeniable that thinner wire diameters have been shown to have the effect of reducing the maximum cutting force in our previous study [4], so wire diameters have a greater effect on the internal fracture of the tissue than on the surface fracture.

### Practical Significance

In previous surgical cases, researchers have typically judged the superiority of commercial snare products by comparing complete resection rates. For example, a snare with a thinner wire diameter and a smaller snare loop can achieve a higher complete resection rate [25, 26]. However, the use of commercial snares has the limitation of not being able to isolate the influencing factors. The new model and evaluation indexes can optimize different single attributes in terms of both easier fracture and achieving a clearer cut margin, which is more intuitive and effective compared to the complete resection rate.

Higher RMS height indicates that the material surface experienced significant friction and tearing during cutting, rather than a clean fracture. Analogizing this physical phenomenon to actual CSP surgery also holds clinical significance. Previous studies have confirmed that when polyps are difficult to cut, forced stretching can cause tissue tearing, resulting in fragmented and unclear resection margins, which severely hinder the accurate assessment of complete tumor resection [27, 28]. This is consistent with the surface fracture phenomenon we proposed. Consequently, the lower RMS values observed in this study suggest clearer margins in clinical applications, thereby aiding pathological evaluation. Although it is indirect evidence based on a physical model, the mechanism basically aligns with existing clinical histological studies, offering strong theoretical support for the clinical translation potential of these findings.

### Limitations and Prospects

Although silicone rubber can simulate the macroscopic fracture behavior of soft tissue, it still has some differences from real biological tissue in terms of fracture mechanisms due to the absence of collagen fibers and multilayered structures [27, 28]. The fracture model and evaluation indexes proposed in this study aim to optimize the cutting capability of the tool, so further validation using real biological tissue remains essential before practical application.

Future work will focus on the optimization of the cutting ability of snare-type tools. Based on the proposed evaluation index, the snare tool geometry and material properties will be parameterized, and experiments will be performed on the optimized tools using biological soft tissues to evaluate the cutting performance in real surgery.

## Data Availability

The experimental data and the simulation results that support the findings of this study are available in Dryad with the identifier 10.5061/dryad.3n5tb2rvz. (Reviewer URL: http://datadryad.org/share/KejFT3xTpMalK5t5phnkm4wY0oRbWfett4NMDcNBoxY).
